# Implementation of hospital antimicrobial stewardship programmes in low- and middle-income countries: a qualitative study from a multi-professional perspective in the Global-PPS network

**DOI:** 10.1186/s13756-025-01541-6

**Published:** 2025-04-05

**Authors:** Ines Pauwels, Ann Versporten, Diane Ashiru-Oredope, Silvia Figueiredo Costa, Herberth Maldonado, Ana Paula Matos Porto, Shaheen Mehtar, Herman Goossens, Sibyl Anthierens, Erika Vlieghe

**Affiliations:** 1 Department of Family Medicine and Population Health, Faculty of Medicine and Health Sciences, University of Antwerp, Antwerp, Belgium; 2 Laboratory of Medical Microbiology, Vaccine & Infectious Disease Institute, Faculty of Medicine and Health Sciences, University of Antwerp, Antwerp, Belgium; 3 Antimicrobial Resistance (AMR) and Healthcare-Associated Infection (HCAI) Division, United Kingdom Health Security Agency (UKHSA), London, UK; 4 School of Pharmacy, University of Nottingham, Nottingham, UK; 5 Centres for Antimicrobial Optimisation Network (CAMO-Net) Brazil, Faculty of Medicine, University of São Paulo, São Paulo, Brazil; 6 Department of Infectious Diseases, Faculty of Medicine, University of São Paulo, São Paulo, Brazil; 7 Departamento de Infectologia e Medicina Tropical, Faculdade de Medicina da Universidade de São Paulo, São Paulo, Brazil; 8 Centro de Estudios en Salud, Universidad del Valle de Guatemala, Guatemala City, Guatemala; 9 Unidad de Cirugía Cardiovascular de Guatemala, Guatemala City, Guatemala; 10 Infection Control Africa Network, Cape Town, South Africa; 11 Infection Control Technical Working Group of the Ministerial Advisory Committee on AMR, Cape Town, South Africa; 12 Unit for Infection Prevention and Control (UIPC), Faculty of Health Sciences, Stellenbosch University, Cape Town, South Africa; 13 Department of General Internal Medicine, Infectious Diseases and Tropical Medicine, University Hospital Antwerp, Antwerp, Belgium

**Keywords:** Antibiotics, Antimicrobial resistance, Antimicrobial stewardship, Hospital, Low- and middle-income countries, Qualitative research

## Abstract

**Background:**

Hospitals in low- and middle-income countries (LMIC) face context-specific challenges in implementing antimicrobial stewardship (AMS) programmes. The Global Point Prevalence Survey (Global-PPS) project has established a network of hospitals across 90 countries, using point prevalence surveys to monitor antimicrobial use and guide AMS activities. However, little is known about AMS implementation in these hospitals. Using qualitative research, we aim to explore the implementation process in LMIC hospitals within the Global-PPS network and the factors influencing it, identify potential implementation strategies, and evaluate the role of Global-PPS in this process.

**Methods:**

A qualitative study was conducted using semi-structured online interviews with healthcare workers (HCWs) involved in AMS in LMIC hospitals within the Global-PPS network. Participants were selected using a combination of convenience and purposive sampling and included clinicians, microbiologists, pharmacists, and nurses. Interviews followed a topic guide based on the integrated checklist of determinants of practice (TICD Checklist). Transcripts were analysed using a combination of inductive and deductive thematic analyses.

**Findings:**

Twenty-two HCWs from 16 countries were interviewed. Hospitals were in different stages of the AMS implementation process at the time of the study, from pre-implementation to institutionalisation of AMS as part of the continuous quality improvement process. While the Global-PPS provided a valuable tool for education and implementation, contextual barriers often hindered the translation of findings into targeted interventions. Four themes influenced AMS implementation, “institutional support and resource allocation”, “AMS team functioning, roles, and expertise”, “adoption and integration of AMS recommendations”, and “data-driven decision-making” as a cross-cutting theme. Key determinants included AMS team competencies, multidisciplinary teams, sustainable funding and leadership support, diagnostic capacity, and reliable data to inform interventions. We also identified various strategies employed by local AMS teams to enhance implementation.

**Conclusions:**

This study examines AMS implementation in LMIC hospitals in the Global-PPS network and identifies key determinants. AMS teams address challenges through task shifting, local engagement and ownership. While empirical evidence on the effectiveness of these strategies is limited, these insights can guide future AMS interventions and studies within LMIC hospitals. Strengthening AMS requires bridging the gap between measurement and action and expanding research on behaviour change.

**Supplementary Information:**

The online version contains supplementary material available at 10.1186/s13756-025-01541-6.

## Background

Antimicrobial resistance (AMR) is a global public health challenge, disproportionately affecting low- and middle-income countries (LMIC) [1]. Optimising antimicrobial use through antimicrobial stewardship (AMS) programmes in healthcare institutions is an essential part of the global AMR response, along with adequate sanitation and infection prevention measures, surveillance, educational activities, research and innovation [2]. Although the core elements of hospital AMS programmes (ASPs) are well-documented, there is no one-size-fits-all approach to integrating these elements into a successful AMS strategy [3–5]. In LMICs, many healthcare institutions face specific challenges, such as a high infectious disease burden, limited access to quality-assured antibiotics, insufficient infection prevention and control infrastructures and a lack of diagnostic capacity to guide clinical decision-making [6, 7]. To effectively address inappropriate antimicrobial prescribing practices, AMS must be tailored to the local context, leveraging existing structures and considering local barriers and facilitators to implementation [8, 9]. This requires a systematic behaviour change approach, with local AMS teams identifying which behaviours need to be changed, analysing the determinants of these behaviours, and selecting interventions suited to this specific context [10]. 

The Global Point Prevalence Survey of Antimicrobial Consumption and Resistance (Global-PPS) project provides a tool to survey antimicrobial prescribing in healthcare settings across the globe using the method of a point prevalence survey (PPS) [11]. Since the start of the project, a global network has been created with healthcare institutions in over 90 countries across low-, middle-, and high-income settings collecting PPS data on antimicrobial use [12]. However, little was known about the AMS implementation process in these hospitals or how Global-PPS influenced this process. To address this gap, an online survey was distributed within the Global-PPS network in 2019, showing substantial variation in AMS implementation among participating institutions. While the survey provided high-level insights, more in-depth research was needed to better understand the context of these hospitals and investigate how local AMS teams could be supported in moving from antimicrobial use data to sustainable quality improvement [13]. This qualitative study, therefore, aimed to explore the AMS implementation process in LMIC hospitals within the Global-PPS network. More specifically, we sought to investigate determinants (barriers and facilitators) of AMS implementation, identify potential strategies to overcome implementation challenges and evaluate the role of Global-PPS in this context.

## Methods

### Study design and setting

This qualitative study, based on online semi-structured interviews with healthcare workers (HCWs), was conducted within the context of the Global-PPS project. While the Global-PPS project’s routine activities are focused on surveying antimicrobial use in hospitals, this study was a separate, one-time investigation carried out in a subset of hospitals within the network. Ethical approval for this study was granted separately by the Ethical Committee of the University Hospital of Antwerp (project ID 3205, 09/05/2022).

### Recruitment and selection

To be eligible for the study, participants had to meet the following criteria: (1) HCWs working in a hospital in a low- or middle-income country (as defined by the 2023 World Bank country classification [14]), (2) involved in the hospital’s AMS activities (both in leadership and operational roles), and (3) having conducted Global-PPS at least once in their institution.

Participants were selected using a two-step sampling process. First, invitations to participate in the study were sent to all respondents from a 2019 survey on the role of Global-PPS in ASPs who met the eligibility criteria (convenience sampling) [13]. In the second step, additional eligible participants from the wider Global-PPS network were invited beyond the original ASP survey respondents (purposive sampling). This was done to obtain a broader diversity in geographical regions, socioeconomic settings, and professional groups among study participants. The email invitation included a study information leaflet, and participants were given the opportunity to ask questions by email. All HCWs who agreed to participate in the study received an informed consent form, and a date and time for the interview were scheduled.

### Data collection

A topic guide was designed based on the study objectives (Additional file 1). To explore the determinants of AMS implementation, the comprehensive, integrated checklist of determinants of practice (TICD Checklist), developed by Flottorp et al. for the ‘Tailored Implementation for Chronic Diseases’ project, was used to guide questions and prompts related to barriers and facilitators [15]. The guide was refined through discussions within the research team and pilot-tested with three European HCWs working in the field of AMS, who had also conducted Global-PPS in their hospital (one pharmacist, one infectious diseases specialist, and one infection control specialist). Minor adaptations were made throughout data collection based on insights from earlier interviews. One PhD researcher, with a background in pharmacy and trained in qualitative research methods (IP), conducted the interviews with the guidance of two senior researchers with expertise in qualitative research, clinical practice and AMS implementation (SA & EV). Interviews were conducted online and recorded through Microsoft Teams. All participants provided verbal informed consent before the start of the interview. All interviews were conducted in English except for one, which was conducted in French. Recordings were pseudonymised and transcribed verbatim. Field notes were made during each interview.

### Data analysis

Interviews were analysed using a hybrid deductive-inductive thematic analysis [16]. First, we developed a framework based on the study objectives, using the TICD checklist to structure the section on implementation determinants [15, 17]. One researcher (IP) coded the data deductively within this framework. Concurrent inductive coding allowed for the creation of novel themes and subthemes not captured within the TICD checklist. These new themes were integrated with the TICD domains through an iterative coding and analysis process. Trustworthiness was ensured by applying validation strategies based on Lincoln and Guba’s criteria [18]. Data collection continued until data saturation was reached, meaning no new themes were identified from additional interviews. During this process, the core research team, comprising researchers with a background in clinical practice, implementation science and qualitative research, discussed findings, reviewed themes, and adjusted the framework as needed (investigator triangulation). Peer debriefing was conducted within the broader international research team to refine interpretations. We documented the analytical process using NVivo (version R1) and developed a codebook. Researcher reflexivity was ensured through reflective memos, documenting team discussions, systematic cross-checking of coding, and transparent theme development.

## Findings

Invitations to participate in the study were sent to 82 eligible participants (70 contacts in the first recruitment phase and 12 additional contacts in the second phase). We received two refusals (one due to time constraints and one due to a change in professional position) and 58 non-responses. Nineteen (27.1%) participants responded positively in the first recruitment phase, and three (25%) HCWs agreed to participate in the second phase. Data saturation was reached at this point, and no further participants were recruited. In total, 22 HCWs working at 21 secondary and tertiary hospitals across 16 countries were interviewed between June 2022 and April 2023 (Table 1, Additional file 2). For two hospitals, individual interviews were conducted with multiple AMS team members with different roles (two participants from one hospital and three from the other). Furthermore, two participants worked at two hospital sites and referred to their respective experiences in both hospitals during the interview. All participants had conducted at least one PPS on antimicrobial prescribing in their hospital as part of the Global-PPS project. Ten participants held senior leadership roles, such as heading the AMS or infection prevention and control (IPC) committee. Five combined leadership with operational AMS tasks (e.g. AMS team coordinator), while seven participants had mainly operational roles (e.g., data collection, ward rounds, prescription monitoring). Interviews were between 36 and 67 min long, with a mean duration of 50 min.

**Table 1:** Participant characteristics

Participant characteristics (*N* = 22)	
**Age in years**,** mean (range)**	46 (36–64)
**Years of professional experience**,** mean (range)**	17 (6–35)
**Gender**,** n (%)**	
Male	12 (54.5)
Female	10 (45.5)
**Profession**,** n (%)**	
Clinical microbiologist	8 (36.4)
Infectious diseases specialist	7 (31.8)
Pharmacist/pharmacologist	3 (13.6)
Other*	4 (18.2)
**Region**, n (%)**	
Africa	8 (36.4)
Asia	9 (40.9)
LatinAmerica	3 (13.6)
Europe	2 (9.1)
**World Bank country classification 2023**,** n (%)**	
Low-income country	4 (18.2)
Lower-middle-income country	12 (54.5)
Upper-middle-income country	6 (27.3)
**Hospital type– complexity of care**,** n (%)**	
Tertiary	16 (72.7)
Secondary	5 (22.7)
Tertiary (paediatric)	1 (4.5)
**Hospital type– financing and organisation**,** n (%)**	
Private†	9 (40.9)
Public	13 (59.1)

* Including: nurse, infection control officer, clinician, epidemiologist

** Countries listed in additional file 2

† Including for-profit and non-profit hospitals

### AMS implementation determinants

**Fig. 1:**
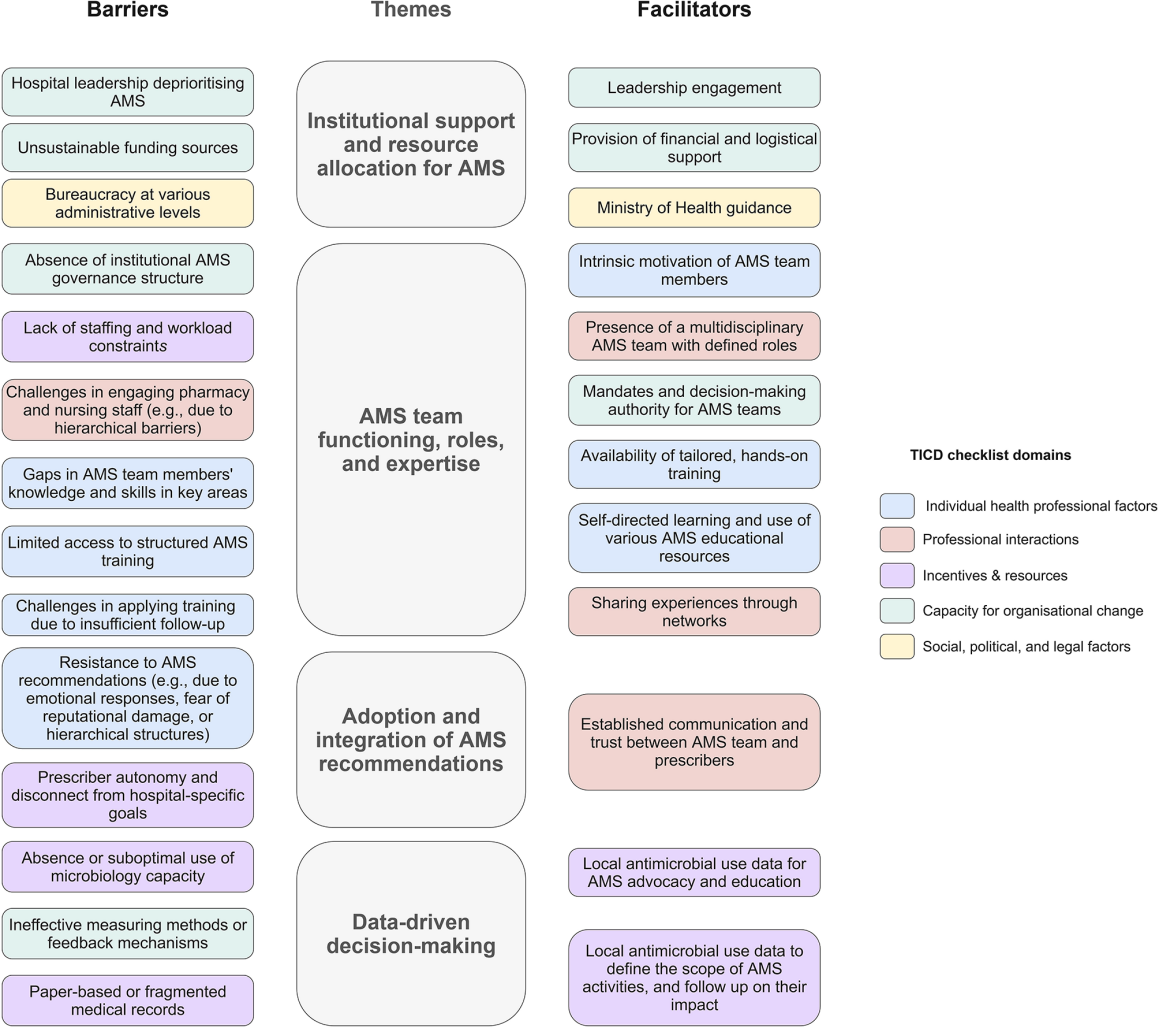
Key determinants of AMS implementation in participating hospitals AMS: antimicrobial stewardship, TICD: ‘Tailored Implementation for Chronic Diseases’ checklist of determinants of practice

Factors influencing the implementation process at the HCWs’ institution were identified from all seven TICD checklist domains. These determinants presented as a dynamic continuum of barriers and facilitators rather than as isolated factors and clustered around four overarching themes: (1) institutional support and resource allocation, (2) AMS team functioning, roles, and expertise, (3) adoption and integration of AMS recommendations, and (4) data-driven decision-making. A complete list of all identified determinants is available in Additional file 3. The following section will describe the 26 key determinants that substantially impacted the implementation process in participating hospitals (Fig. 1).

#### Theme 1: institutional support and resource allocation for AMS

Support from hospital leadership was a key facilitator through allocating resources, providing mandates and authority to AMS teams, active participation in AMS meetings, and assisting with conflict resolution. In other settings, deprioritisation of AMS by hospital leadership was a barrier. Participants noted that leadership was often reluctant to invest in ASPs, as they perceived cost-saving benefits as long-term rather than immediate.

I*n my current hospital*,* the administration is very supportive. In my previous hospital*,* we also had a lot of support from the administrators. They themselves were pushing for these things*,* so the task becomes easier. Administrative will and empowering the stewardship committee to act and get into a discussion with the clinicians*,* if needed*,* is very important. (I16*,* clinical microbiologist)*

*They don’t see stewardship generating money physically*,* the way patients pay for services. It doesn’t come from here. So the funds you generate for them from stewardship are indirect*,* you know*,* as in they have to spend less. And the chief executives don’t like that kind of story. They want money directly*,* in cash. (I07*,* clinical microbiologist)*

Some participants relied on external, non-governmental organisations to fund their ASPs, but the sustainability of these sources was often uncertain, leading to disruptions in activities. While the Ministry of Health facilitated hospital ASP implementation in certain countries (e.g. through guideline development), bureaucratic processes at various administrative levels also slowed down the roll-out of ASPs.

*There’s a national stewardship policy*,* which expired*,* I think*,* in 2022. And there’s another one being developed. But there doesn’t seem to be a translation of what national would want to do to the peripheral level because the necessary logistics for the periphery to be effective are not being put in place. (I15*,* ID specialist)*

#### Theme 2: AMS team functioning, roles, and expertise

##### Composition, role and team dynamics

Several factors influenced participants’ engagement in AMS, such as professional background, connections, and personal interest. In settings with designated funding or support systems, HCWs more often engaged in AMS through their professional roles, whereas intrinsic motivation was a key determinant in settings with little support. Not all hospitals had an AMS team, committee, or formal structure. For some participants, being the only individual working on AMS in their institution was a major barrier to stewardship efforts.

*Well*,* I am in infectious diseases… I know exactly what the problems are with antimicrobial resistance. And I know how antimicrobials are used. So*,* it’s something I should do. It’s my job… and there’s no one*,* and there’s nothing*,* that works with you. You do it because you want to do it. (I05*,* ID specialist)*

Where present, AMS committees, stand-alone or embedded in the Infection Prevention and Control or Medicine and Therapeutics Committee, mainly took up an advisory role. The primary driver for implementation were the daily, operational AMS teams. In hospitals with limited support and a more bottom-up approach to AMS, teams operated independently.

*In my hospital*,* there is only the therapeutic committee and the infection prevention and control committee. So*,* there are two main things*,* but these two committees are so broad… The main people working on these committees are the heads of one or multiple departments in the hospital. […] It’s so difficult for them to focus on the actual work. So*,* I would say that these two committees do not function very well so far. (I18*,* epidemiologist)*

AMS activities were commonly led by ID specialists or microbiologists. Other AMS team members included pharmacists, specialists (non-ID, e.g. surgeons), nurses, IPC professionals and epidemiologists. A lack of staffing for daily AMS activities (e.g. audit and feedback, ward rounds) was a barrier, with many HCWs struggling to balance AMS tasks with routine clinical duties. In some training hospitals, ID fellows took on part of these labour-intensive tasks under senior supervision. Multidisciplinary involvement from pharmacy and nursing was considered valuable but challenging due to hierarchical structures, gaps in AMS knowledge, and heavy workloads. Formal mandates and decision-making authority for the AMS teams were key facilitators.

##### Competencies and capacity-building

While the demand for AMS training was high, its availability varied across settings. In places where tailored, hands-on training was accessible, participants noted being able to apply AMS principles more confidently. Most participants, however, had not received formal AMS training and were primarily self-taught, relying on various educational sources. While this approach allowed some to build AMS expertise and share knowledge with colleagues, its effectiveness depended on access to quality resources and individual effort. Participants highlighted the need for sustainable and accessible training opportunities, with practical training and coaching seen as the most impactful.

*Everything I’m doing here*,* I do by myself with the Internet. I teach myself from the Internet*,* then I go back and teach my residents and nurses. (I13*,* clinician)*

Participants identified key competencies needed in their daily practice, among which communication and convincing skills were considered essential (Additional file 4). Gaps in knowledge and skills in key areas, such as developing guidelines, interpreting microbiology results, creating antibiograms, and using data to drive AMS actions, hindered some from advancing AMS. While some had received training in behaviour change techniques, the lack of follow-up limited practical application. HCWs without an infectious disease background sought specialised training in clinical aspects of antimicrobial therapy.

*I attended certificate programs that focus on the pharmacotherapy of infectious diseases. So*,* it’s a responsibility*,* not only to the hospital but also to myself*,* that I need to improve myself. As a pharmacist I have knowledge. But to be honest*,* that’s not enough*,* so I need to find more resources that can help me improve myself so I can be of better service in my hospital. (I12*,* pharmacist)*

Finally, participants noted that being part of an international network like Global-PPS offered opportunities for knowledge exchange, allowing them to learn from other hospitals’ AMS strategies, tackle shared challenges, and collaborate on research.

#### Theme 3: adoption and integration of AMS recommendations

This theme delves into the dynamics between AMS team members and other HCWs, specifically exploring the mechanisms driving acceptance and uptake of recommendations. Resistance from prescribers to AMS recommendations was a key barrier to implementation. Underlying contributing factors were strongly dependent on the context and included prescribers doubting the safety of recommendations, emotional responses such as frustration towards restrictions, hierarchies, concerns over professional reputation and a lack of awareness about the recommendations and their own prescribing practices (Additional file 3).

*I would cite the policy. I would cite the literature. And they still believed that surgical site infections would happen. We were continuously monitoring surgical site infections*,* and it was very low. We keep showing them the data. We have comparative data before and after AMS—strict implementation of the 24-hour-stop. And the data is still the same. I don’t know. It’s just behaviour*,* probably. It’s emotions rather than science. (I11*,* ID specialist)*

While private hospitals typically had more resources for their activities, AMS teams faced specific barriers, such as attending physicians being less receptive to recommendations, as they felt less strongly associated with the hospital and had greater autonomy. Well-established communication and trust between the AMS team and other HCWs was identified as a key facilitator.

*It’s a little bit easier now for me to talk with other people because they know this is my job and I’ve been doing it for many years*,* so the communication is OK. So*,* once you get to do things again and again*,* it helps build confidence. (I14*,* nurse)*

#### Theme 4: Data-driven decision-making

Data-driven decision-making was a recurring theme, spanning all three previous themes. Local microbiology reporting and data on antimicrobial use were essential for AMS teams in securing leadership support, guiding AMS activities, and improving communication with HCWs.

The absence or suboptimal use of microbiology capacity in some settings was a major barrier to AMS implementation, hindering patient care and the development of treatment guidelines. Where local microbiology data were absent, literature from comparable settings was used to inform guidelines, however, uptake was low as prescribers perceived these guidelines as irrelevant to the context.

*The challenge is the lab. We don’t have a microbiology lab here in the hospital. So*,* what I’m doing is building a network to find those who can help come and help us in the hospital build that laboratory. (I13*,* clinician)*

All participants conducted at least one PPS through the Global-PPS project to assess prescribing quality. For some hospitals, this complemented existing activities; for others, it initiated their AMS activities.

The Global-PPS primarily served as a tool for raising awareness and facilitating education. Results were shared during clinical meetings and ward rounds, initiating discussions on appropriate antimicrobial therapy and AMS interventions. The immediate feedback and benchmarking functionality of Global-PPS facilitated conversations with local HCWs. Some participants used these findings to gain support from management, leading to resource allocation, changes in structure like forming AMS committees, and the development of new policies.

*In my hospital*,* I think obstetrics and gynaecology is the best department. They use 90% cefazolin for their surgeries. And they start it very early. However*,* the bad data came from the orthopaedic and the surgery department. So*,* we used that as a target population. After we received the data*,* we did an educational tour. We visited the orthopaedic department and the surgeons to give them lectures about antimicrobial prophylaxis. And we went to obstetrics and gynaecology to give them reinforcement and give them positive compliments that they did very well. (I09*,* ID specialist)*

Implementation-wise, Global-PPS data helped define the scope of AMS activities and introduced participants to concepts like the WHO AWaRe classification and recommendations for preventing surgical site infections.

*It gave us insight into areas. I mean*,* sometimes we have blindness to certain things that are always happening and that you just take for granted. So*,* the truth is*,* it was pinpointing a lot of things that we already might have suspected and other things that we didn’t have on the radar. (I17*,* ID specialist)*

While many hospitals identified improvement areas through Global-PPS, only a few conducted repeated PPS measurements to evaluate the impact of AMS interventions. Some expressed the need for other data collection methods, such as targeted audits, particularly when certain Global-PPS quality indicators did not align with their specific settings or issues. However, many participants struggled to move beyond identifying areas for improvement due to the implementation barriers described above.

Beyond PPSs, some AMS teams used retrospective or prospective audits to assess prescribing quality or monitored the quantity of antimicrobial consumption (AMC) through pharmacy records. These methods were often perceived as more labour-intensive. Challenges also included data retrieval from paper-based or non-integrated record systems. Participants needed guidance on selecting appropriate metrics and indicators, data analysis, interpretation, and presenting data to stakeholders. Feedback mechanisms varied, with some using active communication, like individualised feedback and clinical meetings, while others relied on less impactful methods like automated emails or bulletin boards.

### Implementation strategies

Many determinants were health system factors, which were difficult for local AMS teams to address. While participants often had to organise activities within the constraints of these barriers, they also identified strategies they felt helped them during AMS implementation. (Table 2). Strategies focused on local engagement and ownership, task-shifting, presenting the benefits of AMS and leveraging quality improvement systems. Although accreditation programs that mandated AMS generally helped persuade management to invest in an AMS programme, their effectiveness was limited when designed as checklists rather than promoting integrated activities and sustainable quality improvement.

**Table 2:** Examples of strategies used by local teams to enhance AMS implementation

Strategy	Examples of specific activities
Encouraging engagement and local ownership	• Involving clinical disciplines in the creation of guidelines and AMS implementation • Identifying champions/early adopters among targeted HCWs • Identifying champions among leaders, hospital management
Visualising benefits of ASPs in terms of clinical outcomes	• Gathering, presenting, and discussing existing evidence with targeted HCWs • Conducting local studies and surveillance, if feasible (e.g. surgical site infection surveillance) • Showcasing success stories
Visualising benefits of ASPs in terms of economic impact	• Providing evidence to convince management of the cost-effectiveness of ASPs
Tailoring communication and education to targeted HCWs needs and learning style	• In-person AMS approach, case-based interactions at the interface between clinical disciplines and microbiology or infectious diseases
Leveraging existing systems for quality improvement & patient safety	• Using accreditation systems to obtain support and resources for AMS activities
Task shifting & promoting multidisciplinary collaboration	• Training the available workforce of non-specialised HCWs e.g., nurses and pharmacists, to increase involvement in AMS activities and empowerment to initiate dialogue with prescribers
Establishing a culture of mentorship within the AMS team	• Support for junior AMS team members by more experienced colleagues

AMS: antimicrobial stewardship, ASPs: antimicrobial stewardship programmes, HCWs: healthcare workers

### Stages of AMS implementation

**Fig. 2:**
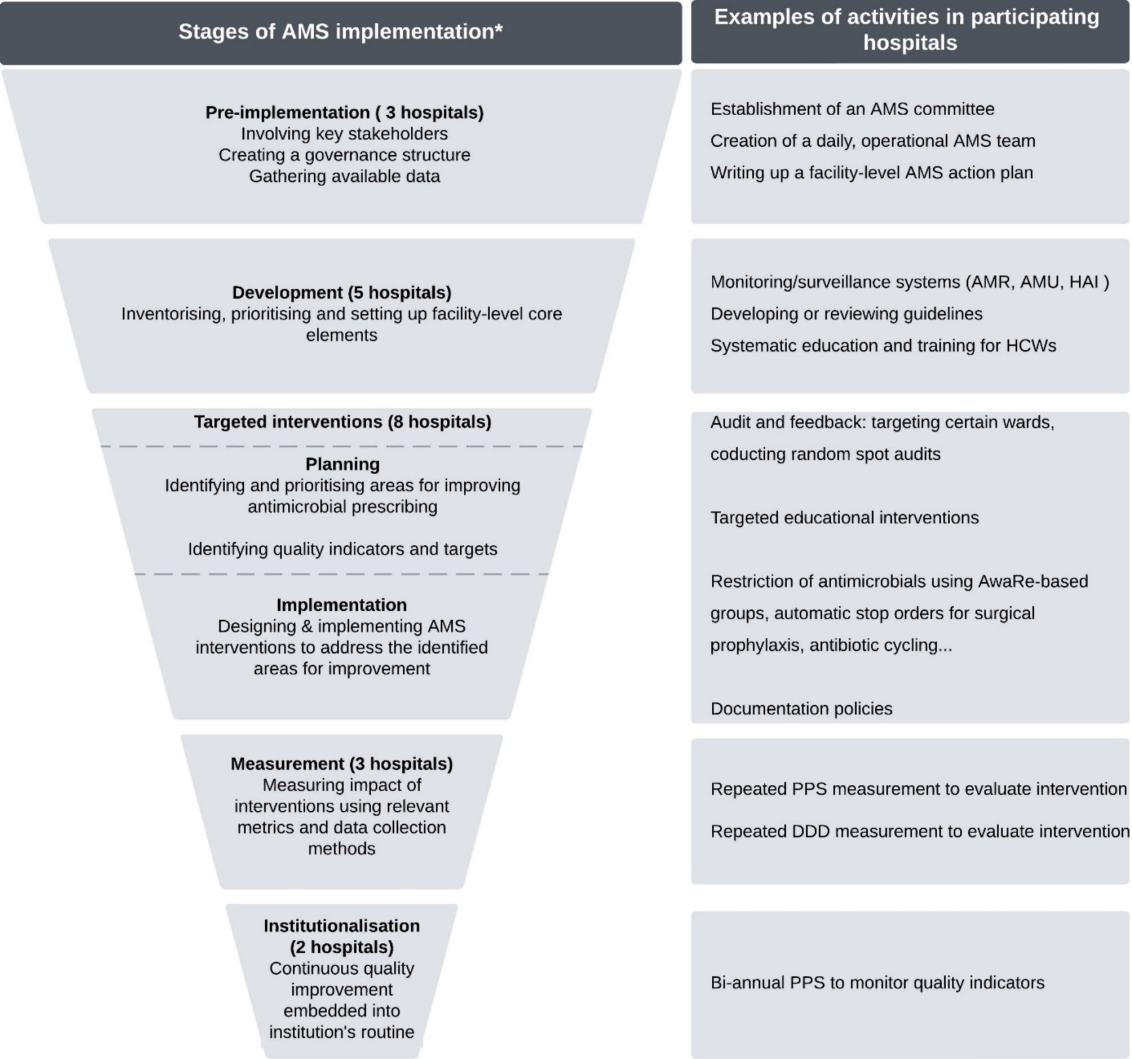
Stages of AMS implementation and examples of activities in participants’ institutions *Stages of implementation are derived from the study data. Each stage shows the number of hospitals at that stage at the time of the study. AMR: antimicrobial resistance, AMS: antimicrobial stewardship, AMU: antimicrobial use, HAI: healthcare-associated infections, HCWs: healthcare workers, PPS: point prevalence survey, DDD: defined daily dose

Hospitals were at different stages of implementation, with fewer progressing through each subsequent stage (Fig. 2).

The importance of the identified determinants varied across different stages of AMS implementation. Hospital support, financial resources, and diagnostic capacity were essential throughout the implementation process. However, their absence posed significant barriers during the early stages. Many participants were still identifying prescribing challenges rather than implementing targeted interventions. Moving forward required a multidisciplinary team, supported by trained staff to facilitate key activities such as audits and feedback.

AMS implementation was further shaped by the competencies of AMS teams, particularly their skills in behaviour change strategies, guideline development and data analysis and interpretation. Many hospitals implementing targeted interventions lacked a structured planning phase or a systematic approach to outcome measurement. Common barriers included suboptimal evaluation methods and difficulties in data retrieval, leaving many without clear evidence of AMS impact.

Key determinants of implementation were broadly consistent across income levels, but severe resource limitations were particularly emphasised in the four Sub-Saharan African low-income countries (LICs). In these settings, implementation efforts relied on short-term funding and were driven by individual motivation and professional networks. No substantial differences were observed between lower-middle-income (L-MICs) and upper-middle-income countries (U-MICs); some well-resourced L-MIC hospitals had progressed further in AMS implementation than certain poorly resourced U-MIC hospitals. In better-resourced hospitals, key challenges and facilitators were more closely related to the adoption and integration of AMS recommendations rather than resource constraints alone.

## Discussion

### AMS implementation determinants and strategies

This study explored the AMS implementation process in a set of hospitals in the Global-PPS network. The heterogeneity of ASPs and the many interrelated determinants underscore the importance of local context and the need for tailored AMS approaches. While many of these determinants are well-documented in the literature, we identified those that had the largest impact on the implementation process and that may be prioritised in the development of future AMS implementation models [8, 9, 19, 20]. 

A lack of sustainable funding and leadership support was a major barrier throughout implementation. Identifying AMS champions among hospital leadership, making a cost-effectiveness business case for AMS, and leveraging accreditation systems, were identified as potential strategies to secure institutional support. Several national and international accreditation schemes do include requirements for infection prevention and AMS [21–23]. However, some participants noted that, in practice, AMS within accreditation processes sometimes remained a checklist exercise rather than leading to integrated activities and sustainable improvement. Given the substantial resources required for accreditation, it is necessary to ensure that these processes move beyond checklists and translate into meaningful, contextually relevant, and feasible improvements [24, 25]. While AMS-specific accreditation programmes exist in certain high-income countries [26–28], the Global Antimicrobial Stewardship Accreditation Scheme (GAMSAS), is now working towards supporting organisations globally establish measurable and sustainable ASPs through accreditation [25, 29]. 

The fact that most key determinants related to the composition, dynamics, and competencies of the operational AMS team highlights the driving force of these teams. Task-shifting was used as a strategy by training pharmacists and nurses to take on active roles. Successful pharmacy-driven ASPs in LMICs have shown the vital roles of clinical pharmacists in audit and feedback, data collection, education, and IPC interventions [30–34]. Similarly, bedside nurses have a critical role in patient monitoring and communication, specimen collection, and prescription review [35]. Incorporating AMS training into curricula and providing continuous education tailored to the needs of the HCW group can strengthen nurses’ and pharmacists’ contributions to AMS [31, 35–37]. Useful approaches to impactful AMS education of non-ID-trained staff include train-the-trainers programmes, mentorship and collaborative learning [31, 32, 38]. However, these initiatives come with challenges related to hierarchies, competencies, and heavy workloads. Hospital leadership, clinicians, and stakeholders must also recognise and support nurses’ and pharmacists’ central role in AMS. Promoting interdisciplinary collaboration is essential to addressing hierarchies and empowering these professionals in their stewardship roles [39–41]. 

Microbiologists play a crucial role in AMS, as shown by their high participation in this study and the various microbiology-led ASPs. However, clinical microbiology services were largely absent in certain hospitals, particularly in Sub-Saharan Africa. This reflects the findings of a recent survey, showing that only 1% of laboratories across 14 Sub-Saharan African countries perform bacterial testing, with 14% of those unable to conduct antimicrobial susceptibility testing [42]. In addition, trained microbiologists are scarce in several LMIC settings; therefore, besides strengthening diagnostic capacity, efforts should be made to make clinical microbiology an attractive career path for young professionals [43]. 

In several of the hospitals, AMS committees or teams were embedded into the hospital’s existing IPC or Medicine and Therapeutics Committees. AMS and IPC are complementary approaches to addressing AMR, and coordinated efforts can lead to greater benefits than each component individually [44, 45]. Making use of available IPC structures and expertise, e.g., in HAI surveillance, could be a valuable strategy in the development of ASPs [46]. 

While the required competencies for AMS teams are well-documented, our findings emphasise the need for training in behaviour change strategies, guideline development, and data-driven planning for ASPs [3, 47]. Despite the recognition of behaviour change approaches in AMS, social science expertise is still lacking within AMS teams [10, 48]. Theoretical frameworks have been developed to guide AMS teams in (1) defining the problem in behavioural terms and evaluating its determinants, (2) designing contextualised interventions to address the behaviour, and (3) evaluating their impact [10, 49–52]. However, the study participants who received training in behaviour change expressed a need for follow-up and guidance to translate theoretical principles into practice. Therefore, efforts should be made to make these frameworks more practical and feasible for those working on AMS implementation. To ensure sustainability, AMS team training can incorporate mentorship and coaching from behaviour change experts, co-creation approaches, as well as train-the-trainer programmes [53, 54]. Meanwhile, the evidence base of behaviour change interventions in AMS in LMIC must also be strengthened to inform policy and contextualised solutions [55]. 

### The role of Global-PPS in AMS activities

This study provides new insights into the use of the Global-PPS in AMS programmes. While previous Global-PPS studies have mainly focused on reporting antimicrobial use patterns, the AMS implementation process was never explored in-depth [11, 56–60]. Global-PPS was found to be a feasible tool to evaluate local prescribing patterns in resource-limited settings, particularly compared to other methods such as prospective audits. While Global-PPS results were primarily used for education and stakeholder engagement, they also helped identify improvement areas and follow-up interventions. However, hospitals still face challenges in turning these findings into effective AMS interventions. Previous studies in LMICs show similar trends, with many using the Global-PPS to measure antimicrobial use but struggling to link it to sustained AMS efforts [61, 62]. These findings also align with a previous survey in the Global-PPS network, highlighting the need for better integration of measurement and intervention in ASPs [13]. Many ASPs did not use a systematic quality improvement approach, making it difficult to assess and improve interventions [3]. To enhance AMS implementation, future efforts should focus on training teams in data collection methods, analysis, interpretation and visualisation. Local teams also need support in using data to identify improvement areas and design targeted, practical interventions. The collaborative nature of the Global-PPS network presents a valuable opportunity for knowledge exchange and sharing of best practices, which could help strengthen AMS programmes across settings.

### Strengths and limitations

This qualitative study provided insights into the perspectives of HCWs involved in AMS in a set of secondary and tertiary care hospitals in U-MIC, L-MIC, and LIC settings. The inclusion of different disciplines yielded rich data, giving insights into the dynamics of the AMS teams. The use of the integrated checklist of determinants of practice (TICD Checklist [15]) during data collection and analysis allowed us to get a broad overview of barriers and facilitators of AMS implementation.

This study had certain limitations. During recruitment, approximately one in four invited HCWs agreed to participate. This could have been due to the sensitive nature of the interview topic, participants’ availability, staff turnover, or language barriers. To mitigate these concerns, we provided clear information about the study, allowed participants to ask questions in advance, offered flexible scheduling, provided the option for interviews in French, and ensured confidentiality through the informed consent process. Despite the recruitment challenges, the study reached data saturation, meaning we found no new themes or insights from additional interviews.

Purposive sampling was used to ensure diversity in geographical regions, socioeconomic settings, and professional groups. However, certain stakeholder voices in AMS, such as nurses, were underrepresented in this study. Similarly, hospital administrators were not included, as participants were HCWs more directly involved in AMS implementation. Given the key role of both nurses and hospital administrators in AMS, this may mean that certain key perspectives were not fully captured. Moreover, determinants relating to acceptance and uptake of recommendations were studied from the perspectives of AMS team members rather than the targeted HCWs. We recognise the value of expanding the scope in future research by including additional stakeholders in the hospital AMS implementation process.

This study was initiated in response to requests from hospitals in the Global-PPS network developing their ASPs. Therefore, the hospitals in this study all had a certain level of AMS implementation. Many participants had prior engagement with the topic, likely shaping their perspectives. The focus was on secondary and tertiary healthcare, excluding primary-level or community healthcare centres, which constitute a significant part of LMIC healthcare settings. Therefore, this study is unrepresentative of the broader LMIC setting. Due to the large heterogeneity in types of hospitals included, and associated health system factors, we were unable to evaluate differences between LIC, L-MIC, and U-MIC settings.

Finally, interviews were conducted by the first author, who was involved in the coordination of the Global-PPS project. While this pre-existing relationship may have influenced participants’ responses, we took deliberate measures to mitigate this. Before and during the interviews, participants were explicitly encouraged to share both positive and critical feedback with the reassurance that honest responses would be valued and would not impact their engagement with the project.

### Implications for practice and future research

Our findings highlight key barriers, such as limited funding and diagnostic capacity, that continue to hinder AMS progress in LMICs. Addressing these barriers must remain a central focus in both practice and future research. Based on our findings, we formulate key areas where AMS efforts can be strengthened to address the realities of healthcare settings in LMICs.

#### Bridging the gap between measurement and action

Research should focus on developing practical models and tools that facilitate data-driven AMS efforts. Strengthening support for AMS teams in translating data into targeted interventions—through training, mentorship, or peer learning—could increase the impact of measurement activities.

#### Rethinking AMS training methods

AMS training should go beyond technical knowledge and provide AMS teams with hands-on problem-solving skills that apply to their specific settings and constraints. Collaborative international projects like Global-PPS offer a valuable platform for shared learning [63]. Initiatives like the drive-AMS project take this further and aim to build capacity by creating local hubs focused on AMS training and expert consultancy [64]. 

#### Expanding the evidence base for behaviour change interventions in LMICs

While our study focused on the implementation determinants of AMS, we also identified certain strategies used by participants, such as task-shifting and mentorship. However, the evidence regarding the impact of these strategies remains limited. Future research should explore which behaviour change strategies are most effective in specific contexts and how they can be practically integrated into AMS practice. This research should aim to identify feasible, context-specific interventions that can be adapted to local healthcare settings. Hub-and-spoke networks or tele-antimicrobial stewardship (TASP) have been suggested as models to facilitate AMS implementation in resource-constrained environments. However, further evaluation of their impact and scalability is needed [65–67]. 

## Conclusions

This study provides insights into the real-world AMS implementation process in hospitals across LMIC settings in the Global-PPS network. Key implementation determinants that may be prioritised in AMS implementation include leadership support, AMS team competencies, sustainable funding, diagnostic capacity, reliable microbiology and antimicrobial use data, and multidisciplinary AMS teams. AMS teams approach these challenges using strategies like task shifting, local engagement and ownership, and leveraging existing systems for quality improvement. Opportunities may lie in bridging the gap between measurement and action, rethinking training methods, and expanding the evidence base for behaviour change approaches to AMS in LMIC hospitals.

## Electronic supplementary material

Below is the link to the electronic supplementary material.

Supplementary Material 1: Topic guide. Topic guide for semi-structured interviews.

Supplementary Material 2: Detailed participant characteristics. Detailed participant characteristics.

Supplementary Material 3: AMS implementation determinants. Description: AMS implementation determinants.

Supplementary Material 4: Competencies for AMS teams. Competency domains for AMS teams identified by interviewed participants

## Data Availability

The datasets supporting this study’s findings are not openly available due to confidentiality guaranteed to participants but are available from the corresponding author upon reasonable request.

## Abbreviations

AMC: Antimicrobial consumption
AMS: Antimicrobial stewardship
ASP: Antimicrobial stewardship programme
AWaRe: Access, Watch, Reserve
GAMSAS: Global Antimicrobial Stewardship Accreditation Scheme
Global-PPS: Global Point Prevalence Survey of Antimicrobial Consumption and Resistance
HCW: Healthcare workers
ID: Infectious diseases
LIC: Low-income countries
LMIC: Low- and middle-income countries
L-MIC: Lower-middle-income countries
U-MIC: Upper-middle-income countries
NGO: Non-governmental organisation
PPS: Point prevalence survey
TASP: Tele-antimicrobial stewardship
TICD: Tailored Implementation for Chronic Diseases
WHO: World Health Organization

## References

[1] Murray CJ, Shunji Ikuta K, Sharara F, Swetschinski L, Robles Aguilar G, Gray A, et al. Global burden of bacterial antimicrobial resistance in 2019: a systematic analysis. Lancet. 2022;399:629–55. 10.1016/S0140-6736(21)02724-0. 35065702 10.1016/S0140-6736(21)02724-0 PMC8841637

[2] World Health Organization. Global Action Plan on Antimicrobial Resistance. 2015. https://www.who.int/publications/i/item/9789241509763. Accessed 12 Apr 2024. 10.7196/samj.9644 26242647

[3] World Health Organization. Antimicrobial stewardship programmes in health-care facilities in low- and middle-income countries. A practical toolkit. 2019. https://www.who.int/publications/i/item/9789241515481. Accessed 9 Jan 2020. 10.1093/jacamr/dlz072 PMC8210188 34222945

[4] Pulcini C, Binda F, Lamkang AS, Trett A, Charani E, Goff DA, et al. Developing core elements and checklist items for global hospital antimicrobial stewardship programmes: a consensus approach. Clin Microbiol Infect. 2019;25:20–5. 10.1016/j.cmi.2018.03.033. 29625170 10.1016/j.cmi.2018.03.033

[5] CDC. Core Elements of Hospital Antibiotic Stewardship Programs. 2019. https://www.cdc.gov/antibiotic-use/core-elements/hospital.html. Accessed 26 Aug 2024.

[6] Cox JA, Vlieghe E, Mendelson M, Wertheim H, Ndegwa L, Villegas MV, et al. Antibiotic stewardship in low- and middle-income countries: the same but different? Clin Microbiol Infect. 2017;23:812–8. 10.1016/j.cmi.2017.07.010. 28712667 10.1016/j.cmi.2017.07.010

[7] Van Dijck C, Vlieghe E, Cox JA. Antibiotic stewardship interventions in hospitals in low-and middle- income countries: a systematic review. Bull World Health Organ. 2018;96:266–80. 10.2471/BLT.17.203448. 29695883 10.2471/BLT.17.203448 PMC5872012

[8] Rolfe R, Kwobah C, Muro F, Ruwanpathirana A, Lyamuya F, Bodinayake C, et al. Barriers to implementing antimicrobial stewardship programs in three low- and middle-income country tertiary care settings: findings from a multi-site qualitative study. Antimicrob Resist Infect Control. 2021;10:60. 10.1186/S13756-021-00929-4. 33766135 10.1186/s13756-021-00929-4 PMC7993456

[9] Fabre V, Secaira C, Cosgrove SE, Lessa FC, Patel TS, Alvarez AA, et al. Deep dive into gaps and barriers to implementation of antimicrobial stewardship programs in hospitals in Latin America. Clin Infect Dis. 2023;77:S53. 10.1093/CID/CIAD184. 37406044 10.1093/cid/ciad184 PMC10321692

[10] Lorencatto F, Charani E, Sevdalis N, Tarrant C, Davey P. Driving sustainable change in antimicrobial prescribing practice: how can social and behavioural sciences help? J Antimicrob Chemother. 2018;73:2613–24. 10.1093/jac/dky222. 30020464 10.1093/jac/dky222

[11] Versporten A, Zarb P, Caniaux I, Gros M-F, Drapier N, Miller M, et al. Antimicrobial consumption and resistance in adult hospital inpatients in 53 countries: results of an internet-based global point prevalence survey. Lancet Glob Health. 2018;6:e619–29. 10.1016/S2214-109X(18)30186-4. 29681513 10.1016/S2214-109X(18)30186-4

[12] Global-PPS. Global Point Prevalence Survey. Impact and Value. 2023. https://www.global-pps.com/documents/. Accessed 12 Jun 2024.

[13] Pauwels I, Versporten A, Vermeulen H, Vlieghe E, Goossens H. Assessing the impact of the global point prevalence survey of antimicrobial consumption and resistance (Global-PPS) on hospital antimicrobial stewardship programmes: results of a worldwide survey. Antimicrob Resist Infect Control. 2021;10:138. 10.1186/s13756-021-01010-w. 34583775 10.1186/s13756-021-01010-w PMC8478001

[14] World Bank Data Help Desk. World Bank Country and Lending Groups 2023. https://datahelpdesk.worldbank.org/knowledgebase/articles/906519-world-bank-country-and-lending-groups. Accessed 31 Jul 2024.

[15] Flottorp SA, Oxman AD, Krause J, Musila NR, Wensing M, Godycki-Cwirko M, et al. A checklist for identifying determinants of practice: A systematic review and synthesis of frameworks and taxonomies of factors that prevent or enable improvements in healthcare professional practice. Implement Sci. 2013;8:35. 10.1186/1748-5908-8-35. 23522377 10.1186/1748-5908-8-35 PMC3617095

[16] Braun V, Clarke V. Using thematic analysis in psychology. Qual Res Psychol. 2006;3:77–101. 10.1191/1478088706QP063OA.

[17] Gale NK, Heath G, Cameron E, Rashid S, Redwood S. Using the framework method for the analysis of qualitative data in multi-disciplinary health research. BMC Med Res Methodol. 2013;13:1–8. 10.1186/1471-2288-13-117. 24047204 10.1186/1471-2288-13-117 PMC3848812

[18] Lincoln Y, Guba E. Naturalistic inquiry. Newbury Park, CA: Sage; 1985.

[19] Harun MGD, Sumon SA, Hasan I, Akther FM, Islam MS, Anwar MMU. Barriers, facilitators, perceptions and impact of interventions in implementing antimicrobial stewardship programs in hospitals of low-middle and middle countries: a scoping review. Antimicrob Resist Infect Control. 2024;13:1–19. 10.1186/S13756-024-01369-6. 38263235 10.1186/s13756-024-01369-6 PMC10804809

[20] Wu S, Tannous E, Haldane V, Ellen ME, Wei X. Barriers and facilitators of implementing interventions to improve appropriate antibiotic use in low- and middle-income countries: a systematic review based on the consolidated framework for implementation research. Implement Sci. 2022;17:30. 10.1186/S13012-022-01209-4. 35550169 10.1186/s13012-022-01209-4 PMC9096759

[21] Organização Nacional de Acreditação (ONA). Instituições Acreditadoras Credenciadas 2025. https://www.ona.org.br/acreditadoras/instituicoes-acreditadoras-credenciadas. Accessed 30 Jan 2025.

[22] National Accreditation Board For Hospitals & Healthcare Providers (NABH). Home 2025. https://nabh.co/. Accessed 30 Jan 2025.

[23] The Joint Commission. Joint Commission Accreditation 2025. https://www.jointcommission.org/what-we-offer/accreditation/. Accessed 30 Jan 2025.

[24] Van Vliet EJ, Soethout J, Churruca K, Braithwaite J, Luxford K, Stewart J, et al. International approaches for implementing accreditation programmes in different healthcare facilities: a comparative case study in Australia, Botswana, Denmark, and Jordan. Int J Qual Health Care. 2023;35:1–12. 10.1093/INTQHC/MZAD026. 10.1093/intqhc/mzad026 37130069

[25] Sneddon J, Drummond F, Guise T, Gilchrist M, Jenkins DR. Accreditation of antimicrobial stewardship programmes: addressing a global need to tackle antimicrobial resistance. JAC Antimicrob Resist. 2023;6. 10.1093/JACAMR/DLAE007. 10.1093/jacamr/dlae007 PMC10833641 38304721

[26] Healthcare Standards Organization. CAN/HSO 5030:2020 - Antimicrobial Stewardship Program 2024. https://healthstandards.org/standard/antimicrobial-stewardship-program/. Accessed 7 Aug 2024.

[27] Infectious Diseases Society of America (IDSA). Antimicrobial Stewardship Centers of Excellence 2024. https://www.idsociety.org/clinical-practice/antimicrobial-stewardship. Accessed 7 Aug 2024.

[28] Australian Commission on Safety and Quality in Health Care. Antimicrobial Stewardship Clinical Care Standard. 2020. https://www.safetyandquality.gov.au/publications-and-resources/resource-library/antimicrobial-stewardship-clinical-care-standard-2020. Accessed 7 Aug 2024.

[29] British Society for Antimicrobial Chemotherapy. GAMSAS 2024. https://ams-accredit.com/. Accessed 7 Aug 2024.

[30] Nampoothiri V, Sangita Sudhir A, Varsha Joseph M, Mohamed Z, Menon V, Charani E, et al. Mapping the implementation of a clinical Pharmacist-Driven antimicrobial stewardship programme at a tertiary care centre in South India. Antibiotics. 2021;10:220. 10.3390/antibiotics10020220. 33672095 10.3390/antibiotics10020220 PMC7926893

[31] Brink AJ, Messina AP, Feldman C, Richards GA, Becker PJ, Goff DA, et al. Antimicrobial stewardship across 47 South African hospitals: an implementation study. Lancet Infect Dis. 2016;16:1017–25. 10.1016/S1473-3099(16)30012-3. 27312577 10.1016/S1473-3099(16)30012-3

[32] Thi Lan Huong V, Thi Dieu Ngan T, Phuong Thao H, Minh Quang L, Thi Thu Hanh T, Thi Hien N, et al. Assessing feasibility of Establishing antimicrobial stewardship programmes in two provincial-level hospitals in Vietnam: an implementation research study. BMJ Open. 2021;11:53343. 10.1136/bmjopen-2021-053343. 10.1136/bmjopen-2021-053343 PMC8488745 34598989

[33] Nyoloka N, Richards C, Mpute W, Chadwala HM, Kumwenda HS, Mwangonde-Phiri V, et al. Pharmacist-Led antimicrobial stewardship programme in two tertiary hospitals in Malawi. Antibiotics. 2024;13:480. 10.3390/ANTIBIOTICS13060480. 38927147 10.3390/antibiotics13060480 PMC11201287

[34] Kerr F, Sefah IA, Essah DO, Cockburn A, Afriyie D, Mahungu J, et al. Practical Pharmacist-Led interventions to improve antimicrobial stewardship in Ghana, Tanzania, Uganda and Zambia. Pharmacy. 2021;9:124. 10.3390/PHARMACY9030124. 34287350 10.3390/pharmacy9030124 PMC8293468

[35] Bos M, Schouten J, De Bot C, Vermeulen H, Hulscher M. A hidden gem in multidisciplinary antimicrobial stewardship: a systematic review on bedside nurses’ activities in daily practice regarding antibiotic use. JAC Antimicrob Resist. 2023;5. 10.1093/JACAMR/DLAD123. 10.1093/jacamr/dlad123 PMC10667038 38021036

[36] Nampoothiri V, Mbamalu O, Mendelson M, Singh S, Charani E. Pharmacist roles in antimicrobial stewardship: a qualitative study from India, South Africa and the united Kingdom. JAC Antimicrob Resist. 2024;6. 10.1093/JACAMR/DLAE047. 10.1093/jacamr/dlae047 PMC11073750 38716399

[37] Chater AM, Family H, Abraao LM, Burnett E, Castro-Sanchez E, Du Toit B, et al. Influences on nurses’ engagement in antimicrobial stewardship behaviours: a multi-country survey using the theoretical domains framework. J Hosp Infect. 2022;129:171–80. 10.1016/J.JHIN.2022.07.010. 35843415 10.1016/j.jhin.2022.07.010

[38] Goff DA, Bauer KA, Brink A, Kolman S, Mendelson M, Messina AP, et al. International train the trainer antibiotic stewardship program for pharmacists: implementation, sustainability, and outcomes. J Am Coll Clin Pharm. 2020;3:869–76. 10.1002/JAC5.1228.

[39] Brink A, Van den Bergh D, Mendelson M, Richards GA. Passing the baton to pharmacists and nurses: new models of antibiotic stewardship for South Africa? South Afr Med J. 2016;106:947. 10.7196/SAMJ.2016.v106i10.11448. 10.7196/SAMJ.2016.v106i10.11448 27725009

[40] Shamas N, Stokle E, Ashiru-Oredope D, Wesangula E. Challenges of implementing antimicrobial stewardship tools in low to middle income countries (LMICs). Infect Prev Pract. 2023;5:100315. 10.1016/j.infpip.2023.100315. 38107237 10.1016/j.infpip.2023.100315 PMC10724472

[41] Charani E, Smith I, Skodvin B, Perozziello A, Lucet J-C, Lescure F-X, et al. Investigating the cultural and contextual determinants of antimicrobial stewardship programmes across low-, middle- and high-income countries—A qualitative study. PLoS ONE. 2019;14:e0209847. 10.1371/journal.pone.0209847. 30650099 10.1371/journal.pone.0209847 PMC6335060

[42] Ondoa P, Kapoor G, Alimi Y, Shumba E, Osena G, Maina M, et al. Bacteriology testing and antimicrobial resistance detection capacity of National tiered laboratory networks in sub-Saharan Africa: an analysis from 14 countries. Lancet Microbe. 2024;6:100976. 10.1016/j.lanmic.2024.100976. 39653051 10.1016/j.lanmic.2024.100976

[43] Wertheim HFL, Huong VTL, Kuijper EJ. Clinical microbiology laboratories in low-resource settings, it is not only about equipment and reagents, but also good governance for sustainability. Clin Microbiol Infect. 2021;27:1389–90. 10.1016/j.cmi.2021.07.027. 34332110 10.1016/j.cmi.2021.07.027

[44] Hassan SK, Dahmash EZ, Madi T, Tarawneh O, Jomhawi T, Alkhob W, et al. Four years after the implementation of antimicrobial stewardship program in Jordan: evaluation of program’s core elements. Front Public Health. 2023;11. 10.3389/fpubh.2023.1078596. 10.3389/fpubh.2023.1078596 PMC10262748 37325334

[45] Baur D, Gladstone BP, Burkert F, Carrara E, Foschi F, Döbele S, et al. Effect of antibiotic stewardship on the incidence of infection and colonisation with antibiotic-resistant bacteria and clostridium difficile infection: a systematic review and meta-analysis. Lancet Infect Dis. 2017;17:990–1001. 10.1016/S1473-3099(17)30325-0. 28629876 10.1016/S1473-3099(17)30325-0

[46] World Health Organization. Improving infection prevention and control at the health facility. Interim practical manual supporting implementation of the WHO Guidelines on Core Components of Infection Prevention and Control Programmes. 2018. https://www.who.int/publications/i/item/WHO-HIS-SDS-2018.10. Accessed 8 Aug 2024.

[47] World Health Organization. WHO competency framework for health workers’ education and training on antimicrobial resistance. 2018. https://www.who.int/publications/i/item/who-competency-framework-for-health-workers%E2%80%99-education-and-training-on-antimicrobial-resistance. Accessed 9 Aug 2024.

[48] Donisi V, Sibani M, Carrara E, Del Piccolo L, Rimondini M, Mazzaferri F, et al. Emotional, cognitive and social factors of antimicrobial prescribing: can antimicrobial stewardship intervention be effective without addressing psycho-social factors? J Antimicrob Chemother. 2019;74:2844–7. 10.1093/JAC/DKZ308. 31299072 10.1093/jac/dkz308

[49] Chadborn T, Williams S, Jorgensen T, Price C, Buckel A, Altieri E. An approach for embedding behavioural science in antimicrobial resistance one health research. J Infect Public Health. 2023;16:134–40. 10.1016/J.JIPH.2023.11.001. 37973498 10.1016/j.jiph.2023.11.001

[50] Cane J, O’Connor D, Michie S. Validation of the theoretical domains framework for use in behaviour change and implementation research. Implement Sci. 2012;7:37. 10.1186/1748-5908-7-37. 22530986 10.1186/1748-5908-7-37 PMC3483008

[51] Michie S, van Stralen MM, West R. The behaviour change wheel: A new method for characterising and designing behaviour change interventions. Implement Sci. 2011;6:42. 10.1186/1748-5908-6-42. 21513547 10.1186/1748-5908-6-42 PMC3096582

[52] World Health Organization Regional Office for Europe. The TAP quick guide. A practical handbook for implementing Tailoring Antimicrobial Resistance Programmes. 2021. https://www.who.int/europe/publications/i/item/9789289055673. Accessed 9 Aug 2024.

[53] Lohiniva AL, Heweidy I, Girgis S, Abouelata O, Ackley C, Samir S, et al. Developing a theory-based behavior change intervention to improve the prescription of surgical prophylaxis. Int J Clin Pharm. 2022;44:227. 10.1007/S11096-021-01338-8. 34800256 10.1007/s11096-021-01338-8 PMC8605786

[54] Radboudumc. Drive-AMS in Low- and Middle Income Countries. 2024. https://www.radboudumc.nl/en/education/courses/dutch-antimicrobial-stewardship/special-programs/drive-ams-in-lmics. Accessed 9 Aug 2024.

[55] Cuevas C, Batura N, Wulandari LPL, Khan M, Wiseman V. Improving antibiotic use through behaviour change: a systematic review of interventions evaluated in low- and middle-income countries. Health Policy Plan. 2021;36:754–73. 10.1093/HEAPOL/CZAB021. 10.1093/heapol/czab021 PMC8488384 33822953

[56] Hsia Y, Lee BR, Versporten A, Yang Y, Bielicki J, Jackson C, et al. Use of the WHO access, watch, and reserve classification to define patterns of hospital antibiotic use (AWaRe): an analysis of paediatric survey data from 56 countries. Lancet Global Health. 2019;7:e861–71. 10.1016/S2214-109X(19)30071-3. 31200888 10.1016/S2214-109X(19)30071-3

[57] Pauwels I, Versporten A, Drapier N, Vlieghe E, Goossens H, Koraqi A, et al. Hospital antibiotic prescribing patterns in adult patients according to the WHO access, watch and reserve classification (AWaRe): results from a worldwide point prevalence survey in 69 countries. J Antimicrob Chemother. 2021;76:1614–24. 10.1093/jac/dkab050. 33822971 10.1093/jac/dkab050 PMC8120336

[58] Porto APM, Goossens H, Versporten A, Costa SF. Global point prevalence survey of antimicrobial consumption in Brazilian hospitals. J Hosp Infect. 2020;104:165–71. 10.1016/J.JHIN.2019.10.016. 31678430 10.1016/j.jhin.2019.10.016

[59] D’Arcy N, Ashiru-Oredope D, Olaoye O, Afriyie D, Akello Z, Ankrah D, et al. Antibiotic prescribing patterns in Ghana, Uganda, Zambia and Tanzania hospitals: results from the global point prevalence survey (G-PPS) on antimicrobial use and stewardship interventions implemented. Antibiotics. 2021;10:1122. 10.3390/ANTIBIOTICS10091122/S1. 34572704 10.3390/antibiotics10091122 PMC8469030

[60] Singh SK, Sengupta S, Antony R, Bhattacharya S, Mukhopadhyay C, Ramasubramanian V, et al. Variations in antibiotic use across India: multi-centre study through global point prevalence survey. J Hosp Infect. 2019;103:280–3. 10.1016/J.JHIN.2019.05.014. 31170422 10.1016/j.jhin.2019.05.014

[61] Saleem Z, Godman B, Cook A, Khan MA, Campbell SM, Seaton RA, et al. Ongoing efforts to improve antimicrobial utilization in hospitals among African countries and implications for the future. Antibiotics. 2022;11:1824. 10.3390/antibiotics11121824. 36551481 10.3390/antibiotics11121824 PMC9774141

[62] Godman B, Egwuenu A, Haque M, Malande OO, Schellack N, Kumar S, et al. Strategies to improve antimicrobial utilization with a special focus on developing countries. Life. 2021;11:528. 10.3390/life11060528. 34200116 10.3390/life11060528 PMC8229985

[63] Tattevin P, Levy Hara G, Toumi A, Enani M, Coombs G, Voss A, et al. Advocacy for increased international efforts for antimicrobial stewardship actions in Low-and Middle-Income countries on behalf of alliance for the prudent use of antimicrobials (APUA), under the auspices of the international society of antimicrobial chemotherapy (ISAC). Front Med. 2020;7:527243. 10.3389/fmed.2020.00503. 10.3389/fmed.2020.00503 PMC7479847 32984380

[64] About drive-AMS. 2024. https://www.global-pps.com/about-ams/. Accessed 20 Aug 2024.

[65] Dirjayanto VJ, Lazarus G, Geraldine P, Dyson NG, Triastari SK, Anjani JV, et al. Efficacy of telemedicine-based antimicrobial stewardship program to combat antimicrobial resistance: A systematic review and meta-analysis. J Telemed Telecare. 2023. 10.1177/1357633X231204919. 37847852 10.1177/1357633X231204919 PMC12095883

[66] Zacchaeus NGP, Palanikumar P, Alexander H, Webster J, Nair IK, Sadanshiv M, et al. Establishing an effective antimicrobial stewardship program at four secondary-care hospitals in India using a hub-and-spoke model. Antimicrob Stewardship Healthc Epidemiol. 2023;3. 10.1017/ASH.2023.171. 10.1017/ash.2023.171 PMC10311688 37396191

[67] Cohn J, Mendelson M, Kanj SS, Shafiq N, Boszczowski I, Laxminarayan R. Accelerating antibiotic access and stewardship: a new model to safeguard public health. Lancet Infect Dis. 2024;24:e584–90. 10.1016/S1473-3099(24)00070-7. 38484749 10.1016/S1473-3099(24)00070-7

