# Supplementary material for: Implementation of hospital antimicrobial stewardship programmes in low- and middle-income countries: a qualitative study from a multi-professional perspective in the Global-PPS network
Source: Antimicrob Resist Infect Control. 2025 Apr 5;14:26. doi: 10.1186/s13756-025-01541-6 (PMC11972458; doi:10.1186/s13756-025-01541-6)
Supplement: Supplementary file 1 — Supplementary Material 1: Topic guide. Topic guide for semi-structured interviews. [file 13756_2025_1541_MOESM1_ESM.pdf]

Implementation of hospital antimicrobial stewardship programmes in low- and middle-income countries: a qualitative study from a multi-professional perspective in the Global-PPS network

Additional file 1. Topic guide

Current antimicrobial stewardship practices

- What is your current position in the hospital?
- How many years have you been working in the hospital?
- Can you tell me what the term 'antimicrobial stewardship' means to you?
- How did you first become involved in antimicrobial stewardship in your hospital? What was the driver for you to get involved in this work?
- Currently, what are your specific tasks and responsibilities in these antimicrobial stewardship activities?
- How do you feel about your current role in antimicrobial stewardship?
    - Prompts: do you feel confident to fulfil your role? Is there anything you would like to change? What could support you?
- Can you tell me what antimicrobial stewardship looks like in your hospital?
    - Prompts: which activities are taking place or are planned? Do you have any prescribing guidelines, restrictions on antibiotics, surveys or audits on antibiotics or other sources of data, training, other activities….

Barriers and enablers for implementation of antimicrobial stewardship

<u>If existing antimicrobial stewardship activities:</u>

- Were there any challenges or difficulties? Where do you think these challenges arise from? How did you address these challenges?
- What has been going well? Which elements have helped you and your team?
    - Prompts for barriers and enablers: use of/access to guidelines, HCW factors (knowledge, skills, attitudes, motivations…), patient factors (demands, beliefs…), incentives , leadership, policies in your organisation or support/legislation at national level.
- What kind of support have you and your team been provided with to help your work on AMS? (e.g. Staff time? Money? Other resources? Commitment from hospital management) How much do you think these resources help you?
- For your work on antimicrobial stewardship, are you collaborating with other healthcare workers in your hospital? Which ones?
- How did you experience working together with other healthcare workers on antimicrobial stewardship?
    - Prompts: What has been going well? What were the challenges/difficulties? How do you feel about the receptiveness of your colleagues to antimicrobial stewardship activities? What do you think would motivate your colleagues to engage in antimicrobial stewardship activities?
- How would you like to see your hospital's stewardship activities evolve in the future?
  Prompts:
    - Are there any stewardship activities that you would like to implement, but haven't been able to implement so far? What is keeping you from implementing them?

    - What would you need to improve the antimicrobial stewardship activities in your hospital? What if all resources were available, how would you get started?

<u>If no antimicrobial stewardship activities:</u>

- According to you, what are the most important reasons why there are no antimicrobial stewardship activities in your hospital?
- What would you need/ do to start antimicrobial stewardship activities? What if (all) resources were available, how would you get started?

Experiences with Global-PPS

The next couple of questions will be about Global-PPS specifically. Please don't hesitate to give your honest and critical opinion. It will help us to further improve Global-PPS as a tool for stewardship.

- How did you experience participating in Global-PPS?
    - Prompts: what went well? Were there any difficulties?
- Would you say the PPS has contributed to your hospital's stewardship activities? In what way or why not?
    - Prompts: did you use the PPS data to initiate/change any stewardship interventions? Did you use the PPS to monitor the impact of these interventions? Why haven't you used these data?
- Is there anything you would change, that would make Global-PPS more useful for stewardship teams?
- Now that you have conducted Global-PPS, what kind of support would you need to turn the data into stewardship actions?

Knowledge and skills needed for stewardship

- What information/training has been offered to you or your colleagues to perform your AMS tasks?
    - Prompts: Has training been mandatory? How helpful have you found this information/training? Who provided this training? How do you keep informed of new guidance?
- Is there any particular <u>knowledge or skills</u> that you feel would help you in your role in AMS? How could this help you?
- What would be the best way to offer these knowledge and skills, for you personally?

- Is there something else you think is relevant to this topic that we did not yet discuss?
