# Supplementary material for: Implementation of hospital antimicrobial stewardship programmes in low- and middle-income countries: a qualitative study from a multi-professional perspective in the Global-PPS network
Source: Antimicrob Resist Infect Control. 2025 Apr 5;14:26. doi: 10.1186/s13756-025-01541-6 (PMC11972458; doi:10.1186/s13756-025-01541-6)
Supplement: Supplementary file 2 — Supplementary Material 2: Detailed participant characteristics. Detailed participant characteristics. [file 13756_2025_1541_MOESM2_ESM.pdf]

Implementation of hospital antimicrobial stewardship programmes in low- and middle-income countries: a qualitative study from a multi-professional perspective in the Global-PPS network

Additional file 2. Detailed participant characteristics

Participant characteristics (n=22)	
**Age in years, mean (range)**	46 (36-64)
**Years of professional experience, mean (range)**	17 (6-35)
**Gender, n (%)**	
Male	12 (54.5)
Female	10 (45.5)
**Profession, n (%)**	
Clinical microbiologist	8 (36.4)
Infectious diseases specialist	7 (31.8)
Pharmacist/pharmacologist	3 (13.6)
Nurse	1 (4.5)
Clinician	1 (4.5)
Epidemiologist	1 (4.5)
Infection control officer	1 (4.5)
**Region and country, n (%)**	
Africa	8 (36.4)
Democratic Republic of the Congo*	1 (4.5)
Ghana	1 (4.5)
Guinea	1 (4.5)
Malawi*	1 (4.5)
Nigeria	3 (13.6)
Uganda*	1 (4.5)
Asia	9 (40.9)
Cambodia	1 (4.5)
India	3 (13.6)
Lao People's Democratic Republic	1 (4.5)
Philippines	3 (13.6)
Thailand	1 (4.5)
Europe	2 (9.1)
Albania	1 (4.5)
Russian Federation	1 (4.5)
Latin-America	3 (13.6)
Argentina	1 (4.5)
Brazil	1 (4.5)
Mexico	1 (4.5)
**World Bank country classification 2023, n (%)**	
Low-income country	4 (18.2)
Lower-middle-income country	12 (54.5)
Upper-middle-income country	6 (27.3)
**Hospital type – complexity of care, n (%)**	
Tertiary	16 (72.7)
Secondary	5 (22.7)
Tertiary (paediatric)	1 (4.5)
**Hospital type – financing and organisation, n (%)**	
Private†	9 (40.9)
Public	13 (59.1)

* Participants enrolled during the second recruitment step.
† Including for-profit and non-profit hospitals.
