# Supplementary material for: Implementation of hospital antimicrobial stewardship programmes in low- and middle-income countries: a qualitative study from a multi-professional perspective in the Global-PPS network
Source: Antimicrob Resist Infect Control. 2025 Apr 5;14:26. doi: 10.1186/s13756-025-01541-6 (PMC11972458; doi:10.1186/s13756-025-01541-6)
Supplement: Supplementary file 3 — Supplementary Material 3: AMS implementation determinants. Description: AMS implementation determinants. [file 13756_2025_1541_MOESM3_ESM.pdf]

Implementation of hospital antimicrobial stewardship programmes in low- and middle-income countries: a qualitative study from a multi-professional perspective in the Global-PPS network

Additional file 3. AMS implementation determinants*

<table>
<tr><th colspan="2">Theme 1: institutional support and resource allocation</th></tr>
<tr><td>Barriers</td><td>Facilitators</td></tr>
<tr><td colspan="2">Incentives and resources</td></tr>
<tr><td></td><td>• AMS is a requirement in accreditation programmes</td></tr>
<tr><td colspan="2">Professional interactions</td></tr>
<tr><td>• Professional hierarchies (e.g., when more junior HCWs lead AMS efforts)</td><td>• Presence of AMS champions among hospital management (e.g., HCWs with background in microbiology in leadership role)</td></tr>
<tr><td colspan="2">Capacity for organisational change</td></tr>
<tr><td>• Hospital leadership deprioritising AMS due to perceived long-term rather than immediate cost-saving benefits
• Depending on non-sustainable sources of (external) funding</td><td>• Leadership engagement through active participation in AMS meetings and decision-making
• Provision of financial and logistical support for AMS
• Accountability of leaders (e.g., department heads)
• External support and collaborations (NGO’s, research institutions)</td></tr>
<tr><td colspan="2">Social, political, legal factors</td></tr>
<tr><td>• Bureaucracy at various administrative levels
• Political instability inhibiting healthcare investments
• Payment policies encouraging antimicrobial prescribing (out-of-pocket payments, pay-for-service models)</td><td>• Ministry of Health supporting implementation, e.g., through development of guidelines and regulations</td></tr>
<tr><th colspan="2">Theme 2: AMS team functioning, roles, and expertise</th></tr>
<tr><td>Barrier</td><td>Facilitator</td></tr>
<tr><td colspan="2">Individual health professional factors</td></tr>
<tr><td>• Gaps in AMS team members' knowledge and skills in key areas
• Limited access to structured AMS training, particularly for non-infectious disease specialists
• Challenges in applying training (e.g., behaviour change techniques) due to insufficient follow-up</td><td>• Availability of hands-on training tailored to the AMS team members’ needs
• Self-directed learning and use of various AMS educational resources
• Intrinsic motivation of AMS team members</td></tr>
<tr><td colspan="2">Professional interactions</td></tr>
<tr><td>• Challenges in engaging pharmacy and nursing staff
• Hierarchy between AMS team members and other HCWs
• Limited connection and communication between AMS team members (e.g., research, microbiology staff) and clinical staff</td><td>• Presence of a multidisciplinary AMS team with defined roles
• Knowledge exchange and collaborations through (international) networks
• Mentorship from senior to junior AMS team members or between institutions
• Interaction between the advisory AMS committee and the AMS team</td></tr>
<tr><td colspan="2">Incentives & resources</td></tr>
<tr><td>• Lack of staffing and workload constraints
• Certain specialties are not yet developed at the facility (e.g., clinical pharmacist or microbiologist)
• Specialised HCWs prefer jobs outside of the hospital for better career opportunities
• Recommended antimicrobials are not available</td><td>• Integrated IT systems facilitate activities such as audit and feedback
• Specialised staff to conduct labour-intensive tasks (e.g., ID fellows)</td></tr>
</table>

Capacity for organisational change	
• **Absence of a formal AMS team or committee leading to isolated efforts**<br>• Disruption of activities due to outbreaks, epidemics, COVID- pandemic	• **Mandates and decision-making authority for AMS teams**
**Theme 3: Adoption and integration of AMS recommendations**	
**Barrier**	**Facilitator**
Guideline factors	
• Guidelines not adapted to or based on local microbiology data<br>• Guidelines are difficult to access, not comprehensive, or format is not adapted to HCWs' needs<br>• Recommended behaviour requires too much effort or disrupts the workflow	• The impact and benefits of adhering to the guideline are visible & measurable<br>• Local ownership in creation of guideline
Individual health professional factors	
• **Resistance to AMS recommendations**<br>• Lack of trust in hospital hygiene/IPC processes<br>• HCWs do not believe in the recommendation outcome<br>• Fear of losing patients<br>• Prescribers are unaware of AMS recommendations or their practice in relation to the recommendations<br>• Limited awareness of AMR among HCWs<br>• Difficulties interpreting antibiogram	• Knowledge of own practice (e.g., through PPS results, audit & feedback)
Patient factors	
• Prescribers feel like they manage complex cases, justifying the use of broad-spectrum, prolonged antibiotics.<br>• Patient demands	
Professional interactions	
• Hierarchies (senior-junior HCWs, between disciplines)	• **Established communication and trust between AMS team and prescribers**<br>• Champions among targeted HCW groups<br>• Representation of ward staff in AMS team
Incentives & resources	
• **Prescriber autonomy and disconnect from hospital-specific goals (e.g., private hospital consultants)**<br>• Difficult work environment for change, (e.g., high workloads/understaffing)	
Social, political, legal factors	
• Concerns over professional reputation	
**Theme 4: Data-driven decision making**	
**Barrier**	**Facilitator**
Incentives & resources	
• **Absence or suboptimal use of microbiology capacity in some settings hinders the development of guidelines and patient care**<br>• **Depending on paper-based or fragmented medical records complicates data retrieval**	• **Local antimicrobial use data for AMS advocacy and education**<br>• **Local antimicrobial use data to define the scope of AMS activities, and follow-up on their impact**
Capacity for organisational change	
• **Ineffective measuring methods or feedback mechanisms**	

*The 26 key determinants significantly influencing the implementation process in participating hospitals are highlighted in bold. AMR: Antimicrobial resistance, AMS: Antimicrobial stewardship, HCWs: Healthcare workers, ID: Infectious diseases, IPC: Infection prevention and control, PPS: Point prevalence survey
