# Supplementary material for: Implementation of hospital antimicrobial stewardship programmes in low- and middle-income countries: a qualitative study from a multi-professional perspective in the Global-PPS network
Source: Antimicrob Resist Infect Control. 2025 Apr 5;14:26. doi: 10.1186/s13756-025-01541-6 (PMC11972458; doi:10.1186/s13756-025-01541-6)
Supplement: Supplementary file 4 — Supplementary Material 4: Competencies for AMS teams. Competency domains for AMS teams identified by interviewed participants [file 13756_2025_1541_MOESM4_ESM.pdf]

**Implementation of hospital antimicrobial stewardship programmes in low- and middle-income countries: a qualitative study from a multi-professional perspective in the Global-PPS network**

## Additional file 4. Competencies for AMS teams

| Competency domains for AMS teams identified by interviewed participants: |
|---|
| • Basic knowledge of microbiology, infectious diseases, AMR and antimicrobial therapy<br>• Setting up antimicrobial stewardship (AMS) programmes<br>• Principles of infection prevention and control (IPC)<br>• Principles of behaviour change and psychology<br>• Health system knowledge<br>• Principles of implementation science and quality improvement<br>• Communication and influencing skills<br>• Programme management and strategic planning<br>• Data measurement, analyses, and presentation<br>• Writing evidence-based guidelines<br>• Interpreting microbiology results and creating cumulative antibiograms |
